# Nasal Nanovaccines for SARS-CoV-2 to Address COVID-19

**DOI:** 10.3390/vaccines10030405

**Published:** 2022-03-08

**Authors:** Jialu Huang, Yubo Ding, Jingwei Yao, Minghui Zhang, Yu Zhang, Zhuoyi Xie, Jianhong Zuo

**Affiliations:** 1 The Laboratory of Translational Medicine, Hengyang Medical School, University of South China, Hengyang 421001, China; 20192013110748@stu.usc.edu.cn (J.H.); 20202013110951@stu.usc.edu.cn (M.Z.); 20202013110957@stu.usc.edu.cn (Y.Z.); 20212013111063@stu.usc.edu.cn (Z.X.); 2 Nanhua Hospital Affiliated to University of South China, Hengyang Medical School, University of South China, Hengyang 421002, China; jianhong.zuo@nhfsyy.cn (Y.D.); 20192103111185@stu.usc.edu.cn (J.Y.); 3 The Third Affiliated Hospital of University of South China, Hengyang Medical School, University of South China, Hengyang 421900, China

**Keywords:** COVID-19, nasal vaccination, nanovaccine

## Abstract

COVID-19 is still prevalent around the globe. Although some SARS-CoV-2 vaccines have been distributed to the population, the shortcomings of vaccines and the continuous emergence of SARS-CoV-2 mutant virus strains are a cause for concern. Thus, it is vital to continue to improve vaccines and vaccine delivery methods. One option is nasal vaccination, which is more convenient than injections and does not require a syringe. Additionally, stronger mucosal immunity is produced under nasal vaccination. The easy accessibility of the intranasal route is more advantageous than injection in the context of the COVID-19 pandemic. Nanoparticles have been proven to be suitable delivery vehicles and adjuvants, and different NPs have different advantages. The shortcomings of the SARS-CoV-2 vaccine may be compensated by selecting or modifying different nanoparticles. It travels along the digestive tract to the intestine, where it is presented by GALT, tissue-resident immune cells, and gastrointestinal lymph nodes. Nasal nanovaccines are easy to use, safe, multifunctional, and can be distributed quickly, demonstrating strong prospects as a vaccination method for SARS-CoV-2, SARS-CoV-2 variants, or SARS-CoV-n.

## 1. Introduction

In 2019, a disease, COVID-19, started to break out all over the world. To date, it is still spreading and mutating [1]. COVID-19 is a disease caused by SARS-CoV-2 (or 2019-nCoV) infection. SARS-CoV-2 belongs to the coronavirus family and infects the host mainly through the respiratory tract. It is important to seek methods of preventing and treating COVID-19. Initially, drugs proven to be effective against viruses were research targets for COVID-19 treatment. Remdesivir is an antiviral drug that is effective in reducing the duration of COVID-19 [2,3] and was approved for the treatment of COVID-19 [4]. However, some clinical trials have shown that Remdesivir only reduces recovery time for COVID-19 patients, and that it cannot be used as a treatment [5,6]. It was also found to have many side effects. For example, patients are more prone to hypokalemia, nausea, and other symptoms [7]. Petra Bistrovic et al. [8] reported transient bradycardia in COVID-19 patients treated with Remdesivir (mainly hepatic/hepatobiliary disorders, renal and urinary disorders and cardiovascular disease [9,10,11,12]). Other drugs, such as Hydroxychloroquine, Ribavirin, Favipiravir, Azithromycin, Lopinavir/Ritonavir, etc., have been confirmed to inhibit the infection or replication of the SARS-CoV-2 to a certain extent [13]. However, these drugs either have strong side effects or their efficacy is unsatisfactory [14,15,16,17]. In the absence of appropriate treatment drugs, the development of vaccines has played an essential role in controlling the expansion of the COVID-19 epidemic. The vaccines are rapidly being developed. More than 200 vaccines are under development, including vaccines for injections and mucosal vaccinations. Many vaccines have passed clinical trials, and many people have received vaccines. However, there remain issues concerning the high variability of SARS-CoV-2, the population’s acceptance of vaccination, and the reliability, side effects, and safety of vaccines. These problems are hindering the development of vaccines, and “vaccination hesitancy” is a common obstacle [18,19,20,21,22]. Therefore, it is important to develop a SARS-CoV-2 vaccine that is efficient and safe. This review aims to offer an overview of the state of the application of SARS-CoV-2 vaccines in animals and humans. Based on the SARS-CoV-2 infection and mutation characteristics, the prospects of nasal nanovaccine are emphatically described.

## 2. The Route of Vaccination

### 2.1. Vaccination with Syringe Needle

**Figure 1:**
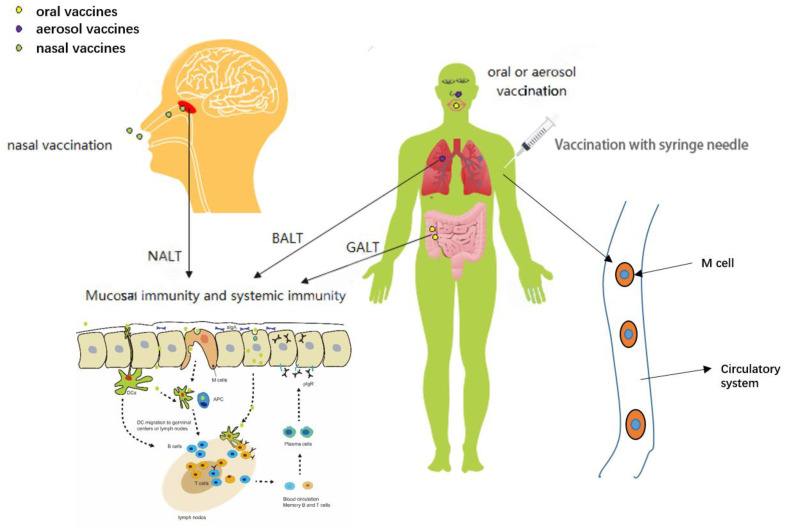
Different routes of vaccination produce different types of immunity at different sites. Oral vaccines along the digestive tract to the GALT, which produce mucosal and systemic immunity. Aerosol vaccines reach BALT by inhalation into the bronchi, which produce mucosal and systemic immunity. Nasal vaccines produce mucosal and systemic immunity at NALT.

There are some types of vaccination that require the use of a syringe, including Subcutaneous, Intradermal and Intramuscular injections (Figure 1) [23]. Intramuscular injection, the traditional and most common means of drug delivery, is also a common form of vaccination. This method of drug delivery is recognized and practiced around the world. However, intramuscular injection is invasive, as the drug or vaccine must be pierced into the skin and muscle by a needle [24]. People usually have relatively low acceptance of intramuscular vaccinations [25]. Additionally, intramuscular injections must be administered by professional medical staff, which limits the efficiency of this vaccination method.

### 2.2. Mucosal Vaccination

Mucosal vaccination, an alternative method of inoculation, includes oral, aerosol and nasal vaccination (Figure 1) [23]. The mucosal immune system (mucosa-associated lymphoid tissues, MALT) defends pathogens from infecting the body via the mucous membranes (the mucosal tissues of the nose, lungs, gastrointestinal tract, vagina, and rectum). Classified by location, it includes nasopharynx-associated lymphoid tissue (NALT), bronchus-associated lymphoid tissue (BALT), and the most distal gut-associated lymphoid tissue (GALT) [26]. Oral vaccines mainly target pathogens by oral-fecal route of transmission. Poliomyelitis vaccine, administered orally, has been successful. It mimics polio infection for better vaccination [27]. Some oral SARS-CoV-2 vaccines are undergoing clinical trials. It travels along the digestive tract to the intestine, where it is presented by GALT, tissue resident immune cells, as well as gastrointestinal lymph nodes. A review of nanostructure-based strategies targeting GALT is discussed in [28]. Resistance to oral vaccines comes from the stomach and intestines. The digestive system is complex, and the vaccine may be destroyed by factors such as gastric acid and protease before reaching GALT [29,30]. Aerosol vaccines, when inhaled, produce mucosal and systemic immunity on BALT in the lungs [31,32,33]. There has been little research and development (Figure 1). SARS-CoV-2 is mainly transmitted through the respiratory tract [34,35,36]. According to the transmission characteristics of SARS-CoV-2, nasal mucosal immunization is an ideal vaccination method for the SARS-CoV-2 vaccine and is theoretically the easiest to obtain [37]. SARS-CoV-2 is readily adsorbed onto the nasal mucosa through nasal inhalation. The nasal mucosa is the first and most important line of defense against SARS-CoV-2 infection. NALT strategically distributed in the nasopharynx and oropharyngeal regions is similarly exposed to the air, and antigens reach NALT through dendritic cells (DCs) or other antigen-presenting cells. Antigens can be collected directly by the synapses of DCs that extend between mucosal epithelial cells [38]. The ideal SARS-CoV-2 vaccines can stimulate the nasal mucosa to produce systemic immunity and mucosal immunity. Among them, the nasal mucosal immune effect evaluation is most important. The spike protein and envelope protein of SARS-CoV-2 can be inhibited by antibodies produced by mucosal immunity, and SARS-CoV-2 in DCs is neutralized [37].

Although intramuscular vaccination is still a common method, nasal vaccination of the SARS-CoV-2 vaccine has become a research trend, and there has been a positive attitude surrounding the development of this type of vaccine.

## 3. The Current Status of Nasal Vaccines in SARS-CoV-2

SARS-CoV-2 nasal vaccines are being developed, and nine of them are in clinical trials. SARS-CoV-2 vaccines include inactivated vaccines, live attenuated vaccines, protein subunit vaccines, nucleic acid vaccines, viral vector-based vaccines, and other vaccines [39]. Inactivated vaccines are prepared using chemical stress or heat stress. In the process of heat stress or chemical stress, inactivated vaccines may lose immunogenicity, so these vaccines often need to be mixed with adjuvants. So far, a total of 28 inactivated vaccines for SARS-CoV-2 have been recorded by the WHO, of which 17 have entered clinical trials, and only one inactivated nasal vaccine has entered a phase I trial (NCT04871737) [40]. Live attenuated vaccines are vaccines made by removing or attenuating parts of the virus. Live attenuated vaccines have appropriate immunogenicity, although live attenuated vaccines require a long time to construct a suitable attenuated virus strain, and the constructed virus strain may undergo virulence reversal at any time. Only eight live attenuated vaccines have been recorded by the WHO. Among the eight vaccines, two nasal vaccines have entered clinical trials (NCT04798001, NCT04619628). Due to the long development time, live attenuated vaccines are not the preferred vaccine for SARS-CoV-2. Viral vector-based vaccines, on the other hand, are vital in the development of SARS-CoV-2 vaccines. This method is to insert antigens into existing successful and safe viral vectors (adenovirus, HIV, etc.) and enter the host through the viral vectors. It is easy for the viral vector to enter the host, causing a stronger host immune response and cross-reaction. In total, 70 viral vector-based vaccines have been recorded by the WHO and 26 viral vector-based vaccines have entered clinical trials, three of which are nasal vaccines (ChiCTR2000037782, discontinued test NCT04679909, NCT04751682, NCT04954287). Nucleic acid vaccines directly introduce the exogenous gene (DNA or RNA), transferring the antigen protein to the host cell to produce the antigen through the expression system of the host cell. The antigens can be recognized by the host immune system to produce anti-SARS-CoV-2 antibodies to achieve the purpose of prevention and treatment. Nucleic acid vaccines can select antigenic determinants by modifying the target gene carried by the gene expression vector. Moreover, the vaccine recipients can benefit from long-term immunity once vaccinated. A total of 76 nucleic acid vaccines have been recorded by the WHO, of which 36 have entered clinical trials. Among them, nasal mRNA vaccines can induce strong mucosal immunity and systemic immunity [41,42]. Protein subunit vaccines are made from one or more immunologically active fragments of SARS-CoV-2. Protein subunit vaccines discard some of the epitopes of SARS-CoV-2, whose immune effect is low and improves the effective antigen utilization efficiency of SARS-CoV-2. It needs to be used together with an adjuvant. In total, 122 protein subunit vaccines have been recorded by the WHO, and 47 have entered clinical trials, including two kinds of nasal protein subunit vaccines (RPCEC0000, IRCT20201214049709N2). All the SARS-CoV-2 vaccines data are from the WHO [23].

Other vaccine platforms recorded include intracellular vaccines, VLP vaccines, and bacterial vector vaccines. The development of SARS-CoV-2 vaccines is diverse, and the development of nasal vaccines is mainly focused on viral vector vaccines and protein subunit vaccines. As a vaccine vector, adenovirus is weakly pathogenic to humans. Even replication-deficient recombinant adenovirus has unapparent side effects on humans and is used as the first choice for nasal vaccines. However, the reason for the failure of a clinical trial of the HIV1 Ad5 vector-based vaccine candidate was existing immunity against the Ad5 vector itself. At the same time, a phase I trial of Ad5-nCoV (a Canadian biotech company) demonstrated reduced vaccine efficacy in individuals with high Ad5 immunity [43]. Viral vector-based vaccines face the possibility of significant inefficiency when they are administered with the same or similar vectors, and the efficacy of human vaccines previously infected with these viruses will also be reduced. The virus vector-based vaccine of the same vector may only be administered once in an individual. This reduces the effectiveness and efficiency of population vaccination. Many nonhomologous viral vectors, such as Newcastle disease virus [40], Poxvirus [44,45,46], etc., can be used as candidates for vaccine vectors. The biosafety of these candidate viruses is still unknown, however, the candidate viral organisms even include uncertain mutations and biosafety of adenoviruses. It is important to consider that the abuse of viral vectors may spell disaster in the future.

The choice of a reliable delivery platform is an important reason to ensure that the vaccine is efficient, safe, and durable. Recently, the delivery platform of nanoparticles (NPs) has seen improvements.

## 4. Nanovaccines

**Figure 2:**
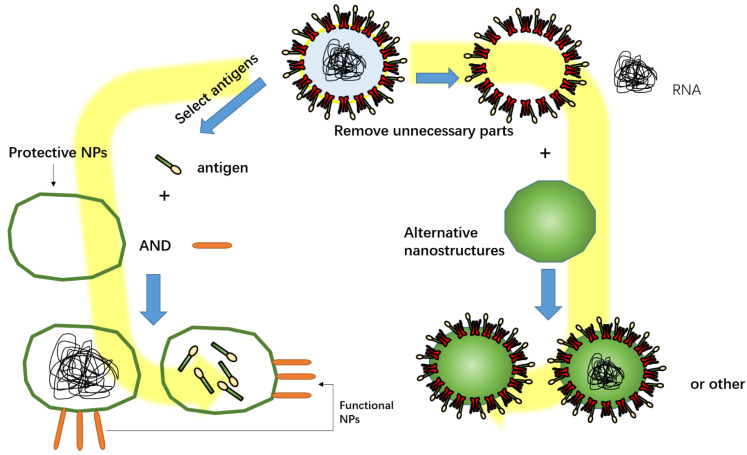
Functional NPs in SARS-CoV-2 vaccines mainly include promoting cell uptake of antigens, protecting antigens, and fully mimicking pathogens. Part of the structure of SARS-CoV-2 was selected to be wrapped in a nanocapsule, or superfluous structures removed from SARS-CoV-2 were replaced with NPs.

A vaccine-based NP delivery vehicle is the inoculum to deliver an antigen in vivo. The nanovaccine has been a novel vaccine delivery platform in recent years [47]. NPs function as an adjuvant to enhance the immune response and the effect of cross-reactivity [48]. Functional NPs in SARS-CoV-2 vaccines mainly include promoting cell uptake of antigens, protecting antigens, and fully mimicking pathogens (like nano-virus) (Figure 2). NPs are mainly divided into four categories: polysaccharide NPs; lipid NPs and protein NPs; Nano-biomimetic delivery vehicles; polymer NPs [49,50].

### 4.1. Polysaccharide Nanoparticles

Polysaccharide nanoparticles belong to a class of natural polymers composed of carbohydrate monomers connected by glycosidic bonds [51]. With inherent immunomodulatory, biocompatibility, biodegradability, low toxicity, and safety characteristics, polysaccharides have attracted much attention in the preparation of nanovaccines and nanomedicine. Polysaccharide adjuvants mainly include chitosan and its derivatives, in addition to glucan, mannan, inulin, and Chinese medicinal herbs.

Chitosan is a cationic polysaccharide biopolymer that exists in the exoskeleton of crustaceans and is produced by acetylation [52]. Chitosan NPs have a large surface area, are capable of the controlled release of drugs, have excellent antibacterial and other biological properties, are non-toxic to humans, and are environmentally friendly and used as a drug delivery vehicle [53,54,55]. Chitosan nanovaccines have proven that the vaccines with chitosan as a carrier can stimulate immune responses in animals [56,57]. In particular, chitosan is soluble in acidic environments and has adhesive properties. The excellent adhesion of chitosan reduces the nasal clearance of the vaccine [58,59,60]. Chitosan can prolong the retention time of drugs or vaccines and improve their efficacy. It has significant advantages as an adjuvant for oral or nasal nanovaccines. Priscila Diniz Lopes et al. [61] confirmed that a chitosan-based IBV-cs vaccine, alone or in combination with a heterologous live attenuated vaccine, can cause humoral and cell-mediated immune responses at the primary site of virus replication and can be localized (the trachea) or in the whole body (kidney) and provide effective protection against IBV infection. Santosh Dhakal et al. [62] confirmed that chitosan NPs improve mucosal immunity and influenza vaccine protection in pigs. Mucosal immune response and systemic immunity are generated after nasal vaccination with chitosan-based nanovaccines. Chitosan NPs are theoretically feasible as the delivery system and adjuvant of SARS-CoV-2 nanovaccines. Adel M. Talaat et al. [63] developed a quil-A-loaded chitosan (QAC) nanovaccine for COVID-19. Neutralizing antibodies and IgA were tested in vaccinated mice (Table 1). The effect of cationic chitosan-based nanovaccines in improving animal humoral immunity is more significant than other chitosan-based nanovaccines [64]. The feasibility of chitosan and its derivatives as SARS-CoV-2 nanovaccine carriers is emphasized in some reviews [65,66]. Chitosan can also be associated with other poly nanoparticles, such as association chitosan-polymers. The associated nanoparticles may be an option in nanovaccine development [67].

### 4.2. Lipid Nanoparticles

#### 4.2.1. Liposomes

Driven by hydrophobicity in water, self-assembled liposomes are spherical vesicles encased by at least a double layer of phospholipids. They are highly fat-soluble and can fuse with cell membranes. Liposome-based vaccines enter the cell by endocytosis. Liposomes were first discovered by Bangham et al. using electron microscopy in the early 1960s [71] and later named “Liposome” by Sessa and Weissmann in 1968 [72]. Generally, liposomes are composed of different types of amphiphilic phospholipids. Combined with other lipids, liposomes can modify the surface characteristics and electrical charge. Liposomes include multilamellar vesicles (MLV), large unilamellar vesicles (LUV), and small unilamellar vesicles (SUV). Gregoriadis et al. [73] have confirmed that liposomes have inherent adjuvant properties. Vaccinated mice produced strong antibody immune responses to the Ags (such as diphtheria toxoid) carried. Moreover, it was found that mice vaccinated with liposome-based vaccines did not have the side effects brought about by conventional vaccine adjuvants, such as granulomas. Most liposomes are negatively charged, and positively charged liposomes composed of positively charged lipids can better adsorb to the nasal mucosa [74]. Ellen K. Wasan et al. [75] intranasally inoculated mice with the L-TriADJ complex coated with cationic liposomes and produced a stronger immune response in mice. Rui Tada et al. [76] found that adhesion of class B CpG ODN to DOTAP/DC-Chol liposomes in nasal vaccine preparation enhances antigen-specific immune responses in mice. Liposomes, especially cationic liposomes, have great potential in the development of SARS-CoV-2 nasal nanovaccines.

#### 4.2.2. Other Lipid Nanoparticles

Liposomes are only an early version of the nanomedicine delivery platform. Many different lipid nanoparticles have been developed, such as solid lipid nanoparticles, lipid nanocapsules and virosomes. These lipid nanoparticles are used in vaccine delivery [50,77,78]. They may provide a direction in the development of nasal nanovaccines for SARS-CoV-2.

### 4.3. Protein Nanoparticles

#### Self-Assembled Proteins

Self-assembled proteins are a higher-level structure made by self-assembly of oligopeptides, nucleotides, and non-biological amphipathic building blocks. To achieve different purposes, researchers have designed different self-assembled proteins. Self-assembled proteins have been widely used in biomolecular engineering and biomedical platforms [79]. In the field of vaccine development, self-assembling proteins can be fused with inactivated pathogens or parts of antigens to produce safe molecular entities that can be effectively delivered to cells to induce immune responses [80]. The development of candidate vaccines based on protein assemblies is a powerful strategy. Ferritin self-assembled NPs are already in clinical trials as nasal nanovaccines [68] (Table 1).

### 4.4. Nano-Biomimetic Delivery Vehicles

Nano-biomimetic delivery vehicles are generally assembled from nanomaterials with a variety of different functions. It is more capable of delivery with nanocarriers synthesized with polymers and lipids [81]. Nano-biomimetic delivery vehicles are made with pathogen antigens into nanovaccines, such as virus-like particles (VLPs), a virus-derived structure composed of one or more different molecules with the ability to self-assemble [82,83]. VLPs mimic the form and size of viruses, however, they lack genetic material, so they have high biological safety due to low infectious doses [84,85]. So far, a series of VLPs candidate vaccines against COVID-19 have been developed, and the effect is being evaluated. Cyrielle Fougerou et al. [86] developed two vaccines based on capsid-like particles (CLP), showing RBD of the SARS-CoV-2 spike protein. Furthermore, the vaccines stimulated strong virus-neutralizing activity in mice. Jing et al. [87] designed a genetic vaccine encoding SARS-CoV-2 virus-like particles. This vaccine induces a strong antiviral-like immune response in mice. Typically, VLPs require nano-biomimetic delivery vehicles in nanovaccines [88]. By improving the charge, size, and other characteristics of VLPs, NPs can better deliver VLPs to the host. Zheng bin et al. [70] designed a nasal nanovaccine, which can induce mucosal immunity by nasal delivery to prevent virus infection. The nanovaccine was composed of poly(I:C) mimicking viral genetic material as adjuvant, biomimetic pulmonary surfactant liposomes as capsid structure of virus and RBDs of SARS-CoV-2 as “spike” to completely simulate the structure of the SARS-CoV-2 (Table 1). NPs may be assembled with antigens to form a SARS-CoV-2-like molecule that mimics the process of viral infection for effective vaccination.

### 4.5. Polymer Nanoparticles

Polymer NPs are nanoparticles formed by the polymerization of one or more organic substances. Poly(D,L-lactic-co-glycolic acid), or PLGA, is the most commonly used synthetic polymer in developing nanoparticle delivery vaccines due to its biodegradability and biocompatibility [89,90]. It was originally used as a suture material for surgery as PLGA is non-toxic and can be degraded into two safe and non-toxic monomers, lactide and glycolide [91,92]. Later, it was found that PLGA functions as an adjuvant and an antigen delivery vehicle. As an antigen delivery vector, PLGA can either encapsulate antigens to form nanocapsules or make antigens adhere to the surface to form nanospheres. The nanocapsules formed by PLGA are similar to liposomal nanovesicles. The pharmacokinetics is regulated by encapsulating the antigen in PLGA particles, and continuous and controlled protein release is allowed to improve the immune response. The sustained release characteristics of PLGA can be used in a single-dose vaccine, which is important for the development of the SARS-CoV-2 vaccine. Some researchers tend to develop single-dose vaccines to achieve rapid vaccination [93,94,95,96,97].

PLGA can also prevent the degradation of antigens. The preservation of antigens is considered by many developers. PLGA-encapsulated vaccines have advantages in antigen protection and can delay the release of antigens. Patki M. et al. [98] found that PLGA loaded with the anti-SARR-CoV-2 drug Remdesivir can continuously and stably release antigen. Qingqin Tan et al. [99] determined that drugs with PLGA as a vector can neutralize a variety of pro-inflammatory cytokines and effectively inhibit the activation of macrophages and neutrophils. Inhibiting inflammation is conducive to reducing the side effects caused by the SARS-CoV-2 vaccine, which means that a nanovaccine with PLGA as a vector is safe. As a nanoparticle, PLGA can provide a characteristic delivery system for antigens and be used as an adjuvant [100,101]. It has great prospects in the development of the SARS-CoV-2 vaccine [102]. In addition to PLGA, other polymer nanoparticles, such as Poly (I:C) as an agonist, also play a similar role [103].

## 5. Combination of Antigen and NPs

Antigens and NPs are generally combined in two ways. The first is to cover the surface of the NPs with antigens. Haptens are not enough to cause recognition by the immune system. The hapten cannot stimulate the body to produce an immune response; thus, the hapten needs to rely on a macromolecular vector [104]. NPs are used as vectors, and antigens are covered on the surface to form a vaccine the same size as the virus to improve its antigenicity. The second way is that the NPs encapsulate the antigen in a vesicle, and this nanovaccine can form a suitable delivery system. Some NPs that are compatible with cell membranes, such as liposomes, cationic NPs, etc., can retain antigens on the cell membrane surface longer and even help the antigen enter the cell. Nanovaccines offer a favorable delivery function, the sustained release of antigens, and the protection of antigens. Incorporating functional NPs can improve the delivery of vaccines. Nanovaccines have diversified functions and diversified design directions, and with different combinations, nanovaccines have different characteristics.

## 6. Nasal Mucosal Immunity of Nanovaccine

**Figure 3:**
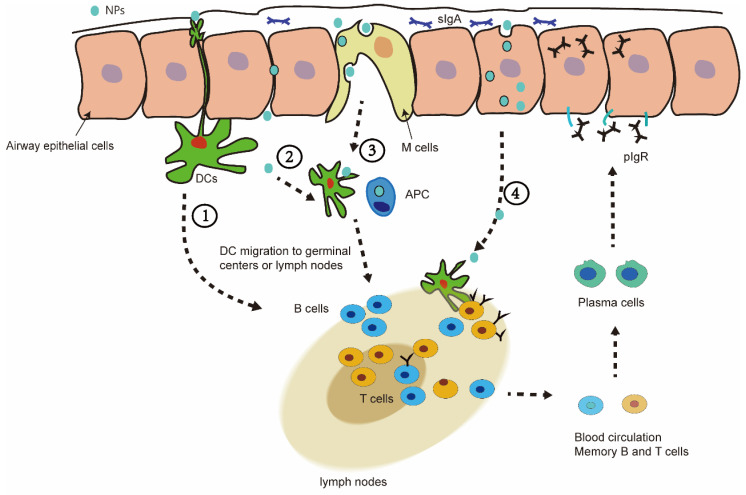
(**1**) Dendritic cells (DC) pass through mucosal epithelial cells to capture NPs in the mucosal layer. (**2**) NPs can also passively permeate through epithelial junctions to access the underlying DC. (**3**) A pocket enriched in APC (macrophages-Mφ, DC, and lymphocytes T) created by the M cells, which perform the sampling of the luminal antigens so that the immune cells contact the NP/antigen. (**4**) NPs can also enter cells through endocytosis and deliver the antigens to cells.

After NPs enter the nasal cavity, they first stay in the mucus, and then pass through the airway epithelial barrier. The stay of the nanovaccine in the mucus is affected by the size of the NPs and other factors. Generally, the 20–80 nm nanovaccine can cause a better immune response in nasal mucosal immunity [105]. Some special NPs can extend the residence time in the nasal mucosa, such as liposomes, chitosan, and so on. Nanovaccines are presented to immune cells in the epithelial cell barrier in different ways. In pathway 1, nanovaccines can be directly captured by dendritic cells through the synapses of the epithelium. In pathway 2, the nanovaccine passively penetrates through the epithelial cell gap and reaches the underlying DC cells. In pathway 3, the vaccine in nanovesicles is captured into the barrier pathway by M cells. In pathway 4, nanovaccines can also enter cells through endocytosis and deliver the antigens to cells (Figure 3) [49,106].

## 7. Why Choose Nasal Nanovaccine in SARS-CoV-2?

### 7.1. Some People Cannot Effectively Resist SARS-CoV-2 after Vaccination

Globally, millions of people have been vaccinated against COVID-19. The vaccines used thus far have passed clinical trials. When COVID-19 re-emerges, part of the vaccinated population will still be infected [107,108,109]. In some people, the SARS-CoV-2 vaccine can only alleviate the symptoms of COVID-19, however, cannot completely resist the invasion of the virus. A study has shown that the mucosal immunity produced by nasal mucosal vaccination of respiratory virus vaccines is more effective in resisting the invasion of respiratory viruses than the systemic immunity produced by injections [110]. Activation of antigen-specific secretion of IgA or sIgA antibodies can prevent pathogens and toxins from adhering to or infecting epithelial cells and destroying the mucosal barrier [111]. IgM and IgG are produced by intramuscular injection, however, IgA can only be produced when high concentrations of IgG are produced. This can protect the lower respiratory tract but not the upper respiratory tract. Nasal immunization can lead to high neutralizing antibody responses and mucosal IgA and T cell responses that almost eradicates SARS-CoV-2 infection in both the upper and lower respiratory tract [112].

Nasal vaccination requires a better vaccine delivery system. Inactivated vaccines, live attenuated vaccines, and viral vector-based vaccines usually produce strong mucosal and systemic immunity following mucosal vaccination. However, the safety of these non-synthetic vectors through human culture and modification is unknown (potential toxicity of proteins, genetics and variation in nucleic acids). As a substitute for these biological vectors, NPs have higher controllability and safety. Protein subunit vaccines tend to be neutralized by antibodies inherent in the mucosal layer, or some proteases present in the nose can also cause their immunogenicity to be reduced [113]. Some NPs, such as PLGA, liposomes or nanoparticle assemblies, prevent antigen neutralization and enhance cellular uptake. As an alternative to vaccine delivery systems or as an additional component of vaccines, NPs may be a better choice.

### 7.2. Troubling Feature: Mutation

The high mutation rate of SARS-CoV-2 is affected by its nucleic acid properties and infection rate. As an RNA virus, its single-stranded structure is not as stable as its double-stranded structure, and it is easy to mutate [114,115]. Strong infectivity can increase the overall mutation rate in SARS-CoV-2. The more the virus replicates, the more mutant strains are produced at the same time. In mutated virus strains, changes in the spike protein cause a greater risk of vaccine ineffectiveness [116,117]. Due to its strong immunogenicity, most researchers have chosen the spike protein as the research target when studying the prevention and treatment of COVID-19. In the phylogenetic tree map of SARS-CoV-2, many variants have appeared, including Delta (B.1.617.2), Alpha (B.1.1.7), Beta (B.1.351), Omicron (B.1.1.529) and Gamma (P.1). These mutant strains all have variant positions in the S gene. However, it is interesting that there are few mutations in the E gene. Thus, the highly conserved E sequence may be a favorable target for vaccine development. We are working on a project related to an S1-E-PLGA nanovaccine, in which we optimize vaccines by reducing ineffective or variable epitopes. The E protein has low immunogenicity. We are trying to improve the vaccine effect through the anti-degradability and adjuvant properties of NPs.

SARS-CoV-2 mutates at an alarming rate. More recently, the Omicron variant has emerged with more genetic mutations (NCBI) [118]. The continuous mutation of SARS-CoV-2 may cause the vaccine to fail. Variants could infect vaccinated people, and the newly established herd immunity could collapse in some areas. The structure of the variant will be re-screened to make a vaccine. However, people would be less receptive to being vaccinated again through a syringe. Inactivated vaccines, live attenuated vaccines, and viral vector-based vaccines also take a long time to develop. It is a better possibility that part of the variant structure and NPs were rapidly assembled for nasal vaccination in dealing with a new pandemic.

## 8. Future and Outlook

COVID-19 continues to spread, and vaccine development and vaccination are ongoing. Many SARS-CoV-2 vaccines face challenges in terms of effectiveness, limitations of vaccination methods, storage requirements, and safety. People have also expressed concern regarding COVID-19 vaccines and many are unwilling to be vaccinated. The nasal vaccine, however, seems to be more acceptable to the public, and the mucosal immunity produced by nasal vaccination can better prevent infection. Nanovaccines have received attention as a new type of vaccine. Nanovaccine technology not only improves the immune effect of antigens, but also ensures the safety of the vaccine. Many kinds of NPs have the function of preventing antigen degradation and sustained release of antigens. Therefore, the development of nanovaccines, especially nasal nanovaccines, appears to have strong prospects.

## Figures and Tables

**Table 1:** Nasal nanovaccines information in COVID-19.

Nasal Candidate Nanovaccines	NPs	Types of NPs	Developers	Functions
A DNA nanovaccine, modified vaccinia ankara expressing SARS-CoV-2 S and N antigens and based with quil-A-loaded chitosan (QAC) [63]	Quil-A-loaded chitosan (QAC)	Polysaccharide	Shaswath et al.	Protection of plasmid integrity and as a adjuvant
A SARS-CoV-2 spike ferritin nanoparticle vaccine (NCT04784767) [68]	Ferritin and Army Liposomal Formulation QS21 (SpFN-ALFQ)	Self-assembled proteins	Kathryn et al.	Enhanced cellular uptake of ferritin and lipidosome NPs, and protection of antigens by liposomes
A Toll-like receptor-4 (TLR4) agonist-based intranasal nanovaccine [69]	inulin acetate (InAc)	Polysaccharide	Kathryn et al.	As toll-like receptor-4 (TLR4) agonist
A inhalable nanovaccine with biomimetic coronavirus structure [70]	poly(I:C) and biomimetic pulmonary surfactant (bio-PS) liposomes	Nano-biomimetic delivery vehicles	Bin Zheng et al.	Completely simulate the structure of the coronavirus

## Data Availability

Not applicable.
